# Tuftsin Combines With Remyelinating Therapy and Improves Outcomes in Models of CNS Demyelinating Disease

**DOI:** 10.3389/fimmu.2018.02784

**Published:** 2018-11-28

**Authors:** Kaitlyn K. Thompson, Jillian C. Nissen, Amanda Pretory, Stella E. Tsirka

**Affiliations:** ^1^Program in Molecular and Cellular Pharmacology, Department of Pharmacological Sciences, Stony Brook University, Stony Brook, NY, United States; ^2^Department of Biological Sciences, State University of New York, College at Old Westbury, Old Westbury, NY, United States

**Keywords:** microglia, anti-inflammatory, tuftsin, remyelination, combination, therapy, autoimmunity

## Abstract

Though promoting remyelination in multiple sclerosis (MS) has emerged as a promising therapeutic strategy, it does not address inflammatory signals that continue to induce neuronal damage and inhibit effectiveness of repair mechanisms. Our lab has previously characterized the immunomodulatory tetrapeptide, tuftsin, which induces an anti-inflammatory shift in microglia and macrophages. This targeted anti-inflammatory agent improves physical deficits in experimental autoimmune encephalomyelitis (EAE), an animal model of MS. Here, we sought to determine whether tuftsin is also effective in combination with benztropine, an FDA-approved drug that stimulates remyelination, in both EAE and in the cuprizone model of demyelination. We show that combining these two agents to promote anti-inflammatory and remyelinating mechanisms alleviates symptoms in EAE and lessens pathological hallmarks in both MS models. Importantly, tuftsin is required to transform the inflammatory CNS environment normally present in EAE/MS into one of an anti-inflammatory nature, and benztropine is required in the cuprizone model to improve remyelination. Our data further support tuftsin's beneficial immunomodulatory activity in the context of EAE, and show that when studying remyelination in the absence of an autoimmune insult, tuftsin still activated microglia toward an anti-inflammatory fate, but benztropine was necessary for significant repair of the damaged myelin. Overall, tuftsin effectively combined with benztropine to significantly improve MS-like pathologies in both models.

## Introduction

Multiple sclerosis (MS) is a chronic, demyelinating autoimmune disease of the central nervous system (CNS) and one of the most common causes of disability among young adults, affecting ~2.5 million people worldwide (1–3). MS is characterized by demyelinated, inflammatory lesions accompanied by blood-brain barrier disruption, usually in the white matter of the brain and spinal cord (1). Within these lesions, both immune and glial cell types actively contribute to neuronal damage. T cells infiltrate the CNS and aberrantly attack the myelin sheath, microglia and blood-derived monocytes promote an inflammatory cascade, and oligodendrocyte precursor cells (OPCs) migrate to these demyelinated, inflammatory sites, but are often unable to differentiate into mature oligodendrocytes (OLs) and thus, are unable to remyelinate the denuded axons (2–5).

Approximately 85% of MS patients are diagnosed with relapsing-remitting MS (RRMS), which consists of acute attacks followed by variable recovery. However, the majority of RRMS patients progress to a subsequent secondary progressive phase (SPMS), in which neurologic disability steadily increases due to failed remyelination and permanent axonal damage (6, 7). Additionally, ~15% of patients are diagnosed with primary progressive MS (PPMS), which consists of continually worsening symptoms from the time of onset (1, 6–8). During these progressive phases, it appears that inflammation diminishes and neurodegeneration predominates due to inefficient CNS plasticity that compensates for already damaged axons and ineffective remyelination and thus, failure to repair and protect naked axons (3, 9).

The current standard therapy for MS consists of long-term use of immunomodulators. Unfortunately, FDA-approved immunomodulatory drugs, such as interferon-β, have only been moderately effective in slowing disease progression and have numerous adverse effects (3, 10). Furthermore, these agents alone have proved incapable of fully preventing disease progression, especially during the phases that consist of continuous, accumulating disability (2). Thus, there is still a dire need for new therapies, both improved immunomodulators and novel repair-facilitating drugs, capable of halting or even reversing disease progression.

An attractive strategy that has gained attention in recent years is to pharmacologically promote remyelination of stripped axons by exploring agents that manipulate cells of the oligodendrocyte lineage. This approach could potentially allow for the return of function to demyelinated neurons and a possible reversal of symptoms. One established method to enhance the remyelinating process in preclinical studies consists of facilitating the differentiation of OPCs into mature OLs capable of myelination (6, 11–13). Specifically, muscarinic receptor antagonism has been shown to be a regulator of OPC maturation, facilitating differentiation into myelin-producing OLs that boost remyelinating mechanisms (14–17).

Though inducing remyelination holds the potential for reversal of disease progression, immunomodulation will remain an important component of treatment to prevent immune-mediated damage and preserve axonal integrity. Microglia and monocyte-derived macrophages contribute in large part to neuronal damage as they can release pro-inflammatory mediators and interact with the adaptive immune system to promote Th1/Th17 pathogenic functions (18, 19). Furthermore, it has been observed in experimental autoimmune encephalomyelitis (EAE), the primary model of MS, that microglial reactivity and contact with axons was associated with axonal degeneration, indicating a direct role for these innate CNS immune cells in neuronal injury (20). Other reports show a primary role for monocyte-derived macrophages in initiating demyelination at EAE onset (21, 22). Thus, targeting both innate immune cell types is a viable and appealing immunotherapeutic strategy to complement a remyelination-enhancing treatment.

We have previously characterized tuftsin, an immunomodulatory tetrapeptide (TKPR), which specifically targets both microglia and macrophages in EAE (23–26). Tuftsin promotes an anti-inflammatory shift in these cells by binding to neuropilin-1 and inducing canonical TGFβ signaling (24, 27). Anti-inflammatory microglia/macrophages are considered more neuroprotective as they release anti-inflammatory cytokines and growth factors, actively phagocytose myelin debris, and promote the anti-inflammatory Th2 response (26) as well as the function of immunosuppressive T-regulatory (Treg) cells (25).

Here, we utilize our microglia/macrophage-targeted tetrapeptide tuftsin in combination with the muscarinic antagonist benztropine. Benztropine is an FDA-approved drug currently in use for treatment of Parkinson's Disease and is also a potent inducer of OPC differentiation (15, 28). It has been reported that benztropine targets OPCs in multiple models of demyelination, including the EAE model, resulting in more mature OLs and reducing demyelination (15). We employ the combination of drugs in two models that recapitulate different aspects of the human disease. We utilize EAE, which primarily captures the inflammatory response in MS, as well as cuprizone, a toxin-induced demyelination model that is useful for examining remyelination mechanisms. In EAE, we observe that tuftsin effectively attenuates CNS inflammation when administered with benztropine, and this combination therapy is successful at improving multiple histopathological hallmarks at multiple time points over the course of the disease. In the cuprizone model, we report that targeting microglia/macrophages with tuftsin alone was not enough to facilitate remyelination and that benztropine was necessary to observe improvements.

## Materials and methods

### Animals

C57BL/6 mice were bred in-house under pathogen-free conditions with controlled temperature and a 12 h light/dark cycle. Access to food and water was *ad libitum*. This study was carried out in accordance with the principles of the Basel Declaration and recommendations of Stony Brook University Institutional Animal Care and Use Committee (SBU IACUC). The protocol was approved by SBU IACUC.

### Induction of EAE with MOG_35−55_ peptide

MOG_35−55_ peptide (MEVGWYRSPFSRVVHLYRNGK) was synthesized by Biomatik (Wilmington, DE). EAE was induced in female mice (8–10 weeks old) by subcutaneous injection on day 0 with 300 μg of MOG_35−55_ thoroughly emulsified in complete Freund's adjuvant (CFA) containing 500 μg of heat-inactivated *Mycobacterium tuberculosis* (Difco, Detroit, MI). Pertussis toxin (List Biologicals, Campbell, CA) was injected intraperitoneally (500 ng dissolved in 200 μL sterile PBS) on days 0 and 2. After immunization with MOG, mice were observed daily and weighed weekly. The severity of disease symptoms was scored on a five-point scale ranging from 0 to 5 with gradations of 0.25 for intermediate symptoms. The score is defined as follows: 0, no detectable symptoms; 1, loss of tail tone; 2, hindlimb weakness or abnormal gait; 3, complete hindlimb paralysis; 4, complete hindlimb paralysis with forelimb weakness or paralysis; 5, moribund or dead. Peak score is defined as the highest score reached during the disease course after the start of drug infusion. Cumulative score is the sum of all scores for the duration of the four-week disease course.

### Cuprizone-induced demyelination

Eight-weeks old female C57BL/6 mice were fed 0.2% (w/w) cuprizone mixed into standard rodent chow (Envigo). The mice were maintained on the cuprizone diet for 5 weeks. At week 4 of cuprizone administration, mice were implanted with mini-osmotic pumps (as described below) containing either saline, tuftsin, or both benztropine and tuftsin. Upon withdrawal of cuprizone at week 5, mice were reverted to standard chow. One week-post cuprizone withdrawal, mice were euthanized and brains were collected, embedded, sectioned, and stained for histological analyses described below.

### Drug delivery

Alzet miniosmotic pumps (Durect, Cupertino, CA) were used for time-controlled drug delivery. Fourteen-day pumps (rate of infusion 0.25 μL/h, 100 μL total volume) were filled with either vehicle or 500 μM tuftsin (Sigma) (23–26), 18 μg/μl benztropine (Sigma), or both tuftsin and benztropine at the same doses and incubated overnight at 37°C before use. The pumps were implanted subcutaneously in the back of the mice under anesthesia 14-days post-MOG immunization. For experiments where tuftsin was administered prior to addition of benztropine, 7-days pumps containing vehicle or tuftsin alone were implanted on Day 14 post-MOG immunization and were replaced with 7-days pumps containing either vehicle or both tuftsin and benztropine at Day 21.

### Tissue collection and processing

Mice were deeply anesthetized with intraperitoneal injection of 2.5% avertin (0.02 mL/g body weight) and transcardially perfused using PBS (pH 7.4) followed by 4% paraformaldehyde (PFA) in PBS (pH 7.4). Spinal cords and brains were isolated, post-fixed in 4% PFA, and dehydrated in 30% sucrose. For spinal cord, the meninges were removed, the lumbar spinal cord was cut into equal sections, embedded in optimal cutting temperature (OCT) compound (Tissue Tek), sectioned onto slides at 20 μm thickness, frozen, and stored at −80°C until use.

### Eriochrome cyanine staining

Eriochrome cyanine (EC) stain was used to visualize myelin in the lumbar region of the spinal cord for EAE animals as we have previously described (26) as well as the corpus callosum in cuprizone-treated animals. Spinal cord or brain sections previously stored at −80°C were air-dried overnight at room temperature. The sections were then incubated at 37°C for 2 h in a dry incubator. After submerging in acetone for 5 min, the slides were air-dried for 30 min and then stained in EC solution (0.2% EC (Sigma), 0.5% H_2_SO_4_ (Sigma), 10% iron alum (Sigma) in distilled water) for 30 min, differentiated in 5% iron alum (Sigma) for 10 min, and placed in borax-ferricyanide solution (1% borax (Sigma), 1.25% potassium ferricyanide (Sigma), in distilled water) for 5 min. The slides were then dehydrated through graded ethanol solutions and coverslipped using SecureMount (Fisher Scientific, NJ, USA). The stained sections were imaged on a Nikon Eclipse E600 microscope at 40 × magnification. ImageJ freeware (NIH) was used to measure the demyelinated and total areas of the white matter. Images were cropped to remove the gray matter regions prior to quantification. To distinguish between positive-staining white matter areas and demyelinated regions, thresholding was used to obtain a binary signal. The demyelinated area was determined by subtracting the myelinated region from the total area, and percentage was calculated as follows: Demyelinated area (%) = [(Demyelinated area in white matter/Total white matter area) × 100]. Six full coronal sections were analyzed for each biological replicate.

### Transmission electron microscopy (TEM)

EAE mice were processed for electron microscopy 21-days post-MOG immunization by intracardial perfusion with 2% PFA/2.5% glutaraldehyde in 0.1 M PBS (pH 7.4). The lumbar region of the spinal cord was isolated, embedded in 3% low melting point agarose gel, and cut into 50 μm sections using a Leica VT-1000 Vibratome. Free-floating sections were post-fixed overnight at 4°C and then placed in 2% osmium tetroxide in 0.1 M phosphate buffer. Sections were washed in 0.1 M phosphate buffer and dehydrated in graded series of ethyl alcohol. The sections were then vacuum-infiltrated in Durcupan ACM embedding agent (Electron Microscopy Sciences) overnight. Ultrathin sections (70–80 nm) were generated using a Reichert-Jung 701704 Ultracut E ultramicrotome and counterstained with uranyl acetate and lead citrate. Samples were viewed with a Tecnai™ Spirit Bio-Twin G^2^ transmission electron microscope (FEI Company) and digital images were acquired at 14,900 × with an AMT XR-60 CCD Digital Camera System (Advanced Microscopy Techniques).

### Immunofluorescence

Spinal cord or brain sections mounted on slides used for immunofluorescence were rinsed in PBS for 5 min to remove residual OCT from the embedding process. After washing, samples were blocked in serum of the host of the secondary antibody (5% serum and 0.3% BSA in PBS with 0.2% Triton-X 100) and then incubated overnight with rabbit anti-mouse Iba1 (1:500, Wako), mouse anti-mouse iNOS (1:500, BD Biosciences), mouse anti-mouse Arg-1 (1:500, BD Biosciences), rabbit anti-mouse NG2 (1:500, a generous gift from the Levine lab), mouse anti-mouse CC1 (1:100, EMD Millipore), or rabbit anti-mouse GST-pi (1:250, MBL International) in 0.3% BSA in PBS with 0.2% Triton-X 100. After washing with PBS, sections were incubated with fluorescence-conjugated Alexa Fluor 488 or 555 goat anti-rabbit or goat anti-mouse antibody for 1 h at room temperature, washed three times with PBS, and mounted using Fluoromount-G with DAPI (Southern Biotech, USA). The sections were imaged at 63 × using a Zeiss LSM 510 confocal microscope. Images were acquired at the same six pre-designated locations along the ventral columns of the lumbar spinal cord section for each biological replicate.

### Morphological analysis

To quantitatively examine microglial morphology, 20 μm z-stacks (taken on a Zeiss LSM 510 confocal microscope at 63 × magnification) of cells positive for Iba1 immunoreactivity in the lumbar spinal cord were imported into Neurolucida software. Cell bodies and processes were traced and a 3D rendering was created. The 3D reconstructions were imported into Neurolucida Explorer for analyses.

### Flow cytometry

Twenty-one days post-MOG immunization, mice were euthanized by saline perfusion. The spinal cord was isolated and digested in papain (1 mg/mL) (Sigma). Following trituration and removal of papain solution, cells underwent density centrifugation in 30% Percoll to remove myelin debris. Cells were rinsed in HBSS twice and then blocked with CD16/32 Fc block (Biolegend, 1:50 in FACS buffer) for 30 min. Cells were then stained with CD11b-APC (Biolegend, 1:100), CD86-Pacific Blue (Biolegend, 1:100), and CD206-PE (Biolegend, 1:100) for 30 min and washed two times with FACS buffer. Samples were analyzed using a BD LSR Fortessa and post-processed on FlowJo software.

### Statistics

For multiple comparisons within a group, statistical analysis was performed using a two-way ANOVA followed by a Bonferroni *post-hoc* test. For comparisons between groups a two-tailed *t*-test was used, as indicated by the figure legends. For all figures, *p* < 0.05 was considered significant and is marked by ^*^; *p* < 0.01 and *p* < 0.001 are marked by ^**^ and ^***^, respectively. All results are represented as the mean with error bars indicating the standard error of the mean (SEM). In all experiments, *n* refers to the number of biological replicates used for each condition.

## Results

### Concomitant therapeutic administration of tuftsin and benztropine reduces the severity of EAE behaviors compared to untreated mice and benztropine alone

Considering that tuftsin is an immunomodulator, an immune-mediated model of MS, EAE, was initially applied to study the combination therapy (29, 30). EAE was induced by MOG immunization in 8–10 weeks old female C57BL/6 mice. Motor dysfunction was monitored daily through a “disease score” measure. Drug delivery using mini-osmotic pumps was initiated at day 14 post-immunization when mice had a score of at least a 0.5, indicating initial loss of tail tone. This served to model a therapeutic treatment regimen, which is more relevant to patient treatment timelines. Mice were then euthanized at either day 21 or 28 for histological analyses.

**Figure 1:**
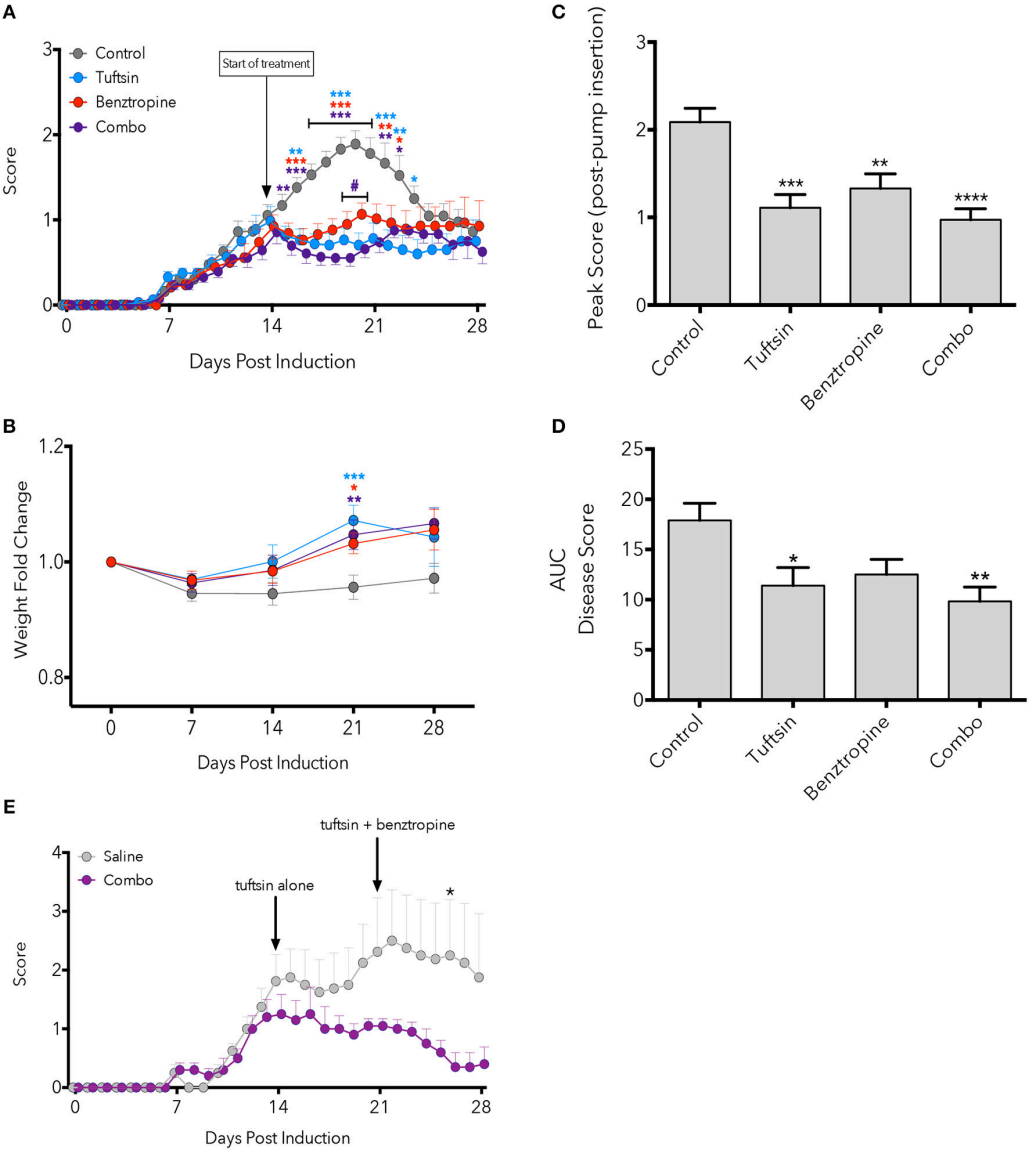
Combinatorial therapy attenuates EAE. EAE was induced by subcutaneous injection of MOG_35−55_ in CFA and i.p. injections of pertussis toxin on Day 0 and 2. At Day 14 post-MOG immunization, a mini-osmotic pump containing tuftsin, benztropine, or both drugs were implanted subcutaneously in treated groups. Mice were euthanized at either day 21 or 28 for histological analysis. Comparisons are between control and tuftsin (blue asterisks), control and benztropine (red asterisks), control and combo (purple asterisks), and benztropine and combo (purple pound sign) **(A)**. Weights were recorded weekly and compared between control and all treated groups with asterisks indicating significance as stated above **(B)**. Weights are plotted as fold change from Day 0 weight. Peak score **(C)** and area under the curve **(D)** were compared between all groups. EAE was induced as in **(**A**)** and mini-osmotic pumps were inserted on Day 14 containing tuftsin alone or saline. Pumps were replaced on Day 21 with benztropine added to treated mice. Arrows denote day of pump insertion and pump replacement **(E)**. All data are mean ± SEM. *n* = 5–29 **(A–D)**, *n* = 4–5 **(E)**, analyzed by two-way ANOVA, *p < 0.05; **p < 0.01, ***p < 0.001, ****p < 0.0001.

Here we observed that monotherapies of either tuftsin or benztropine as well as the combination of the two significantly ameliorated the EAE disease course compared to untreated mice, most notably at the peak of the disease (Figure 1A). Quantification of the area under the curve (an indicator of overall illness throughout the disease course) showed significant differences between control and tuftsin-treated mice, and between control and combo-treated mice (Figure 1B). Therapeutic treatment with the agents together, though not statistically different than either monotherapy, displayed a notably significant decrease from control in the peak score reached by the animals after initiation of drug infusion (Figure 1C). All three modalities reduced weight loss at the day 21 peak of the disease compared to control mice (Figure 1D).

We also evaluated if establishing a more anti-inflammatory CNS environment prior to promoting OPC differentiation would affect the course of EAE. Here, we administered tuftsin alone starting on Day 14 and added benztropine on Day 21. While we observed attenuation of the disease at all time points tested, the decrease was statistically significant only at Day 26, 5 days after the addition of benztropine (Figure 1E).

### Combining therapeutics concurrently reduces demyelination during both the peak and recovery phases of EAE

**Figure 2:**
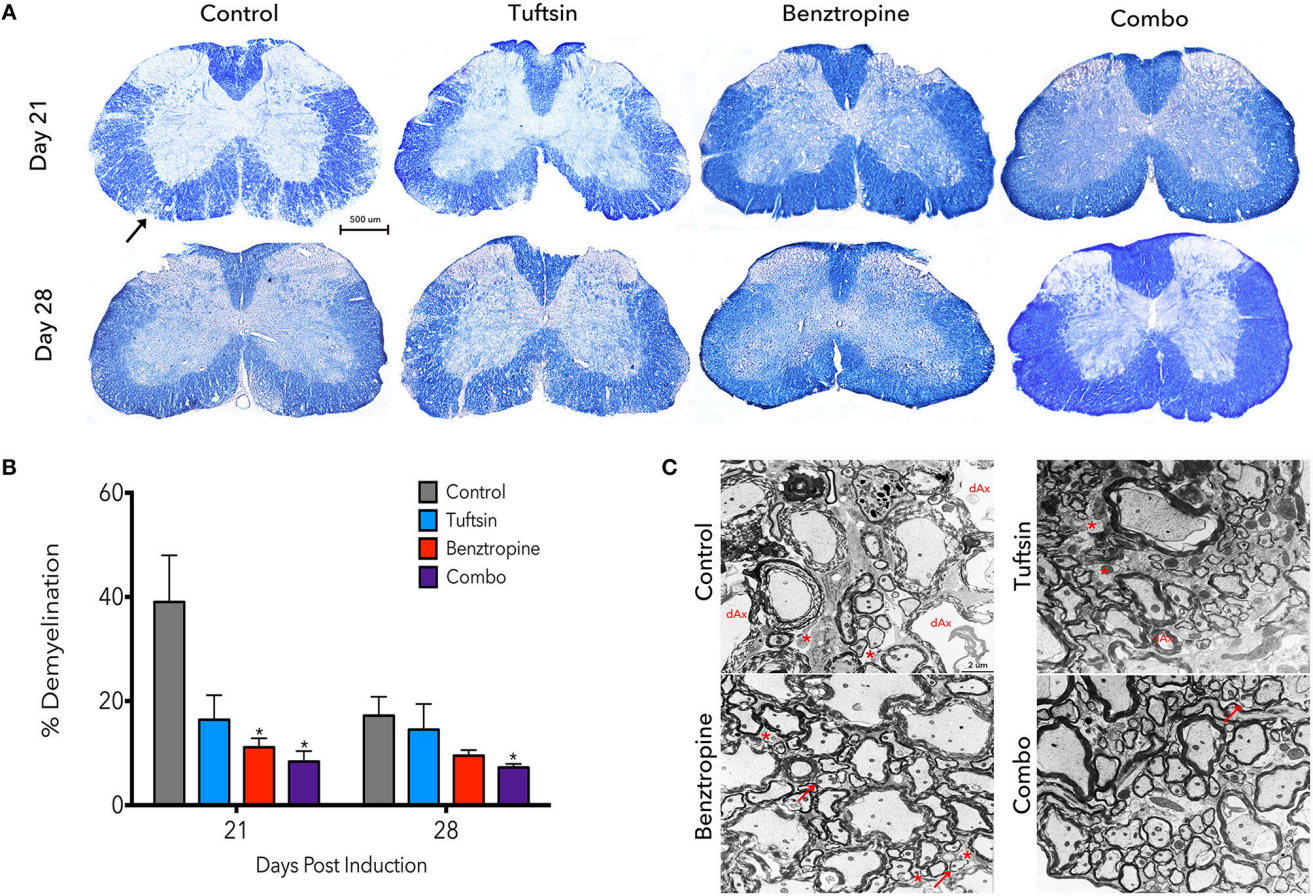
Demyelination is reduced throughout the disease course with a combinatorial treatment strategy. The lumbar spinal cord region was isolated from control, tuftsin-treated, benztropine-treated, or combo-treated mice at days 21 and 28 post-MOG immunization. Eriochrome cyanine stain was used to visualize demyeliation as myelinated regions of the white matter stain dark blue and demyelinated areas are diminished in color. Black arrow shows example of demyelinated area. Images of full coronal sections are shown **(A)**. Demyelinated areas were measured using ImageJ and quantified by subtracting the myelinated area of the white matter from the total area of the white matter and normalizing to total area **(B)**. Data are mean ±SEM. *n* = 5–7, *p < 0.05 compared to control. Scale bar: 500 um. At day 21, myelin was also visualized using transmission electron microscopy. Representative images from the ventral white matter are shown **(C)**. Degenerating axons (dAx) is characterized by diffuse cytoplasmic staining (31). Arrows indicate thin myelin indicative of remyelination. Demyelinated axons are marked by asterisks. Scale bar: 2 um.

Demyelination is a prominent pathological hallmark of MS and is also recapitulated in EAE (17, 30). Demyelination in the lumbar region of the spinal cord was assessed by EC staining at day 21, the peak of the disease, and day 28, during recovery (Figure 2A). Benztropine alone and, to a further degree, the combination of benztropine and tuftsin significantly decreased the percent of demyelination in the white matter compared to untreated EAE mice at the peak of the disease. During recovery, only the combination therapy was effective in significantly decreasing percent demyelination compared to untreated animals (Figure 2B). Thus, unlike the monotherapies, this dual drug treatment regimen resulted in the greatest increase in numbers of myelinated axons compared to untreated mice throughout the duration of the MOG-EAE disease course.

Demyelination was more closely evaluated using electron microscopy at the peak of the disease. Examining the ventral white matter of the lumbar spinal cord region, combination-treated mice revealed intact myelinated axons. Sections from untreated EAE mice, however, exhibited disorganized myelin sheaths with possibly degenerating axons characterized by transparent axoplasm (Figure 2C) (17, 31). Taken together, it appears that these agents complement each other to achieve reduction in demyelination throughout the disease course.

### Benztropine alone induces a shift toward more mature oligodendrocytes at the inflammatory disease peak

**Figure 3:**
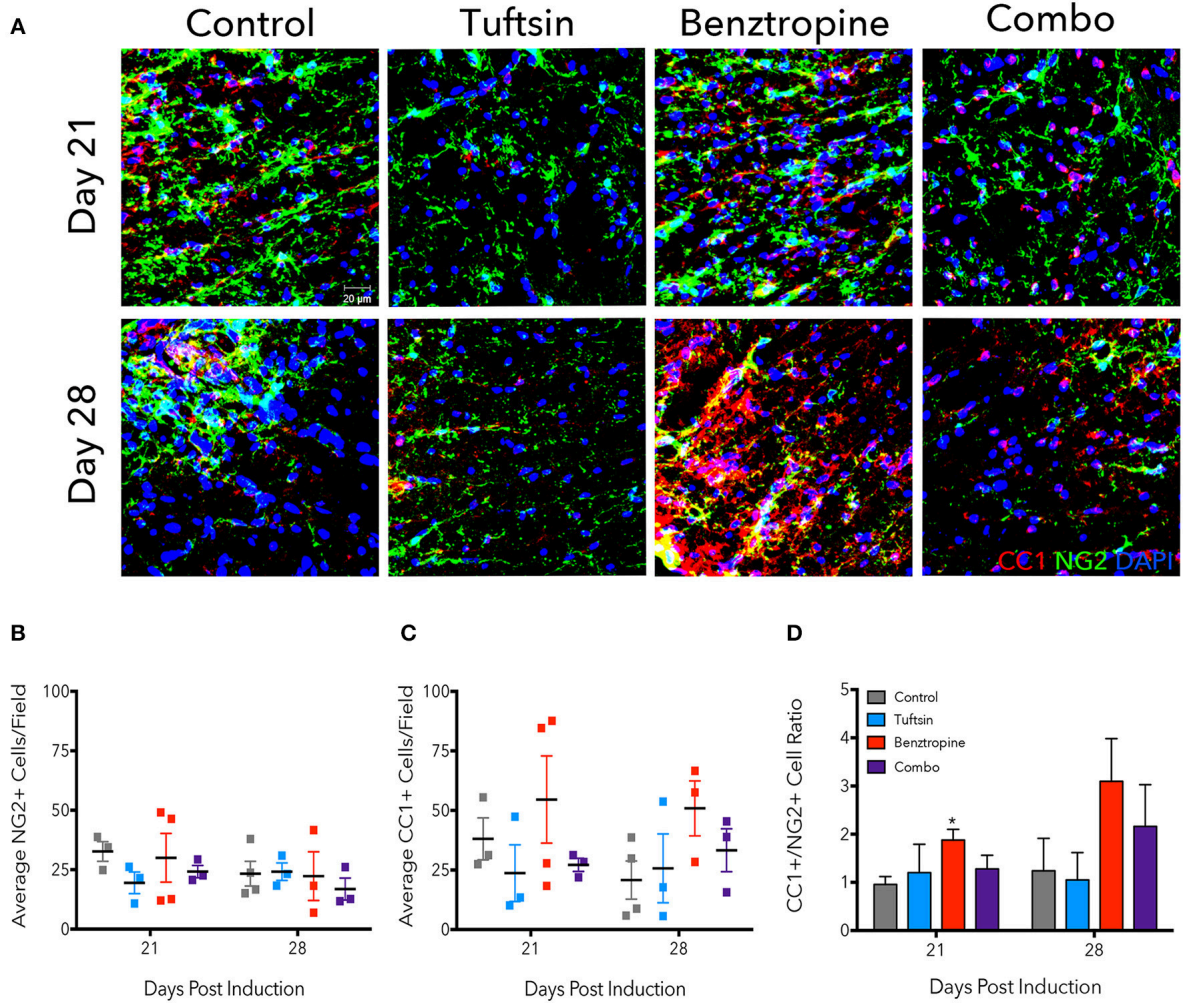
Benztropine induces a shift toward more CC1^+^ oligodendrocytes in EAE. The lumbar spinal cord region was isolated from control and treated mice at days 21 and 28 post-EAE induction. To visualize OPCs and mature OLs, immunostaining for NG2^+^ cells (green) and CC1^+^ cells (red) was performed. Cell nuclei were identified by DAPI (blue) **(A)**. Numbers of NG2^+^ **(B)** and CC1^+^ **(C)** cells were counted per field and ratios of CC1^+^/NG2^+^ cells were calculated **(D)**. Data are mean ± SEM. *n* = 3–4, *p < 0.05 compared to control. Scale bar: 20 um.

Due to significantly less demyelination evident in benztropine- and combination-treated mice, we examined the numbers of immature OPCs and mature OLs, as benztropine has previously been seen to induce a shift in favor of higher OL numbers through promoting OPC differentiation (15). We immunostained lumbar spinal cord tissue with NG2, a marker of OPCs, and CC1, a marker of mature OLs at days 21 and 28 (Figure 3A). We also stained for GST-π as a marker of mature oligodendrocytes (Figure S2). Though no significant differences were found in the average number of NG2^+^ cells/field (Figure 3B), the NG2^+^ cells did exhibit a more reactive phenotype, characterized by increased immunofluorescence intensity and obvious changes in morphology (5), in both control and benztropine-treated mice. There were no significant changes in CC1^+^ cells (Figure 3C), however, it is interesting to note that two of the four benztropine-treated mice analyzed on day 21 had notably higher number of cells/field than the other two animals, and that these same two mice also had more NG2^+^ cells/field as well as more severe EAE scores. This could imply a more intense immune response in these animals and thus, enhanced OPC recruitment. Benztropine is facilitating the differentiation of these recruited OPCs with a significant shift toward more CC1^+^ cells evident by the significant increase in the ratio of CC1^+^/NG2^+^ cells at this time point. No significant shift toward mature CC1^+^ OLs was evident in the combination-treated mice at either time point (Figure 3D).

### Remyelinating therapy alone is not sufficient to induce an anti-inflammatory shift in microglia/macrophages throughout the EAE disease course

Since tuftsin specifically targets microglia and macrophage populations to ease inflammation (23–26), we evaluated the morphology and phenotype of microglia/macrophages in the lumbar region of the spinal cord. Microglia/macrophage activation and recruitment significantly contribute to the severity of EAE (19, 32, 33). Pro-inflammatory activation of these cells is accompanied by dramatic changes in morphology including the retraction of processes, less branching, and swollen cell bodies (34, 35).

**Figure 4:**
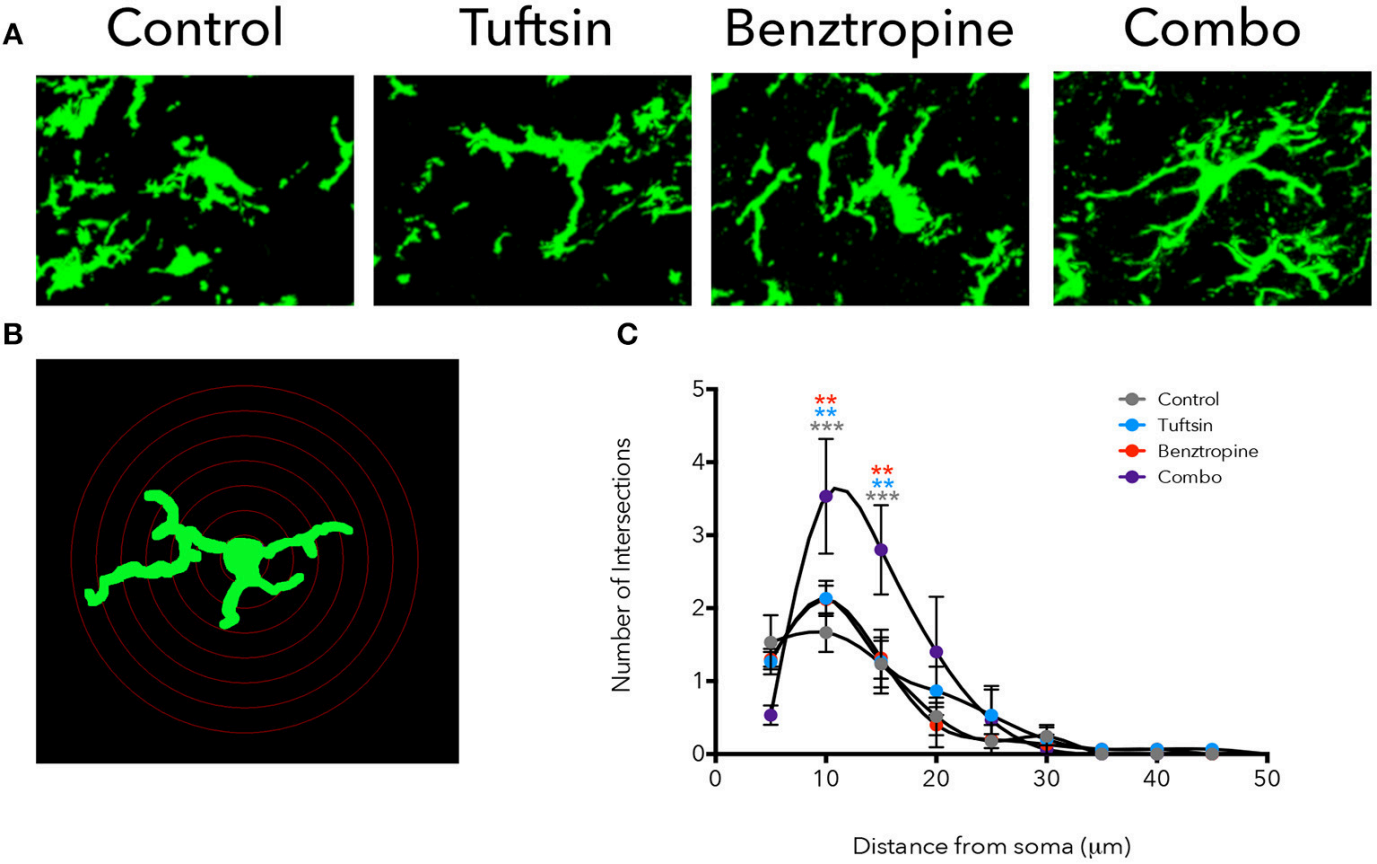
Treatment with tuftsin and benztropine alters complexity of microglial morphology. The lumbar spinal cord was isolated from EAE mice at day 21. To visualize microglia, Iba1 immunostaining (green) was performed and 20 micron z-stacks of the cells were imaged using confocal microscopy **(A)**. Fluorescent images were traced in Neurolucida software and 3D reconstructions from the tracings were analyzed using the linear Sholl analysis with 5 um concentric rings **(B)**. Number of intersections at each distance from the soma was averaged from 5 cells/animal and plotted **(C)**. Comparisons are between control and combination (gray asterisks), tuftsin and combination (blue asterisks), and benztropine and combination (red asterisks). Data are mean ± SEM. *n* = 3 animals, 5 cells/animal analyzed, **p < 0.01, ***p < 0.001.

**Figure 5:**
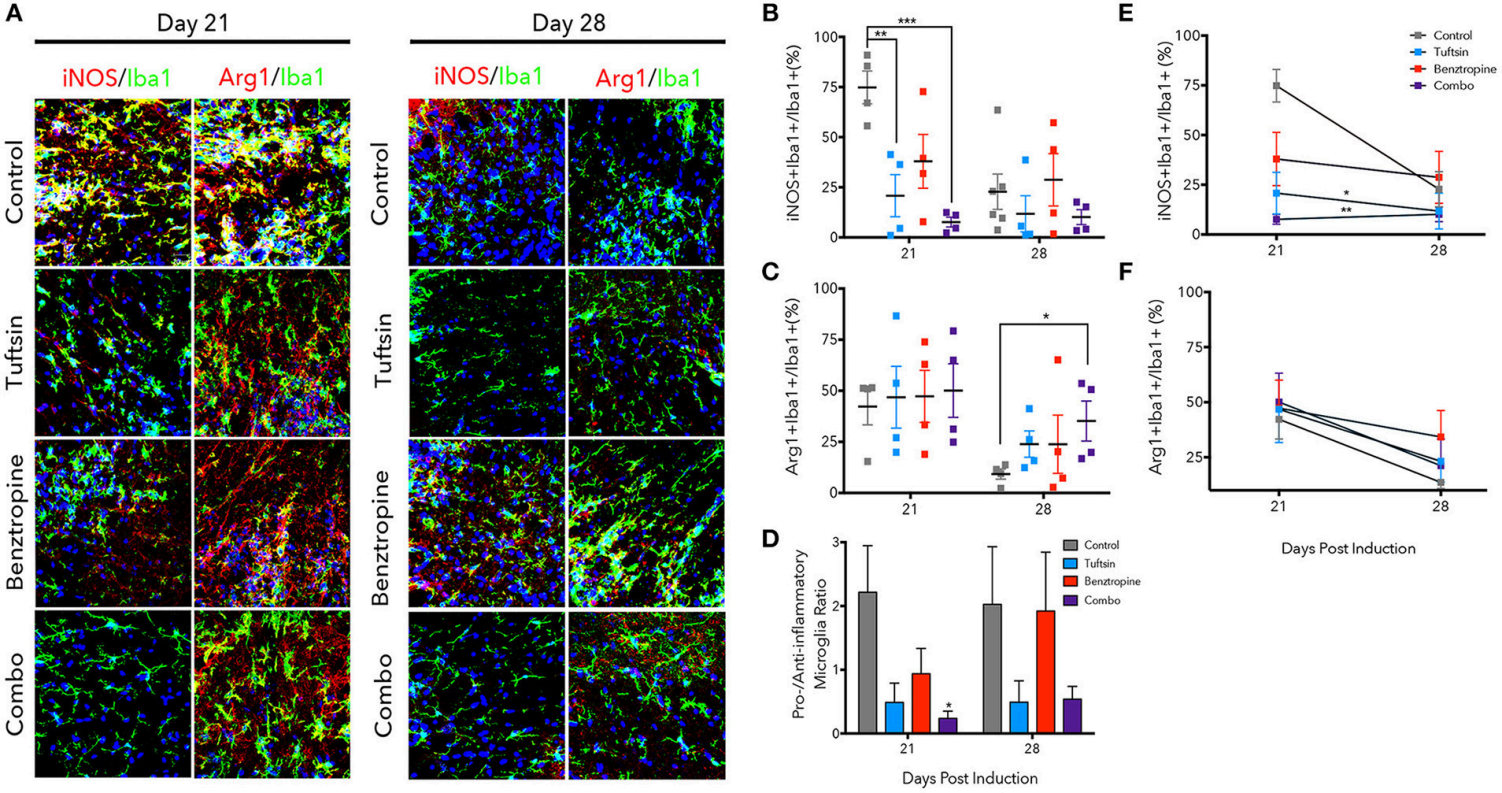
Tuftsin, alone and together with benztropine, dampens inflammation in EAE. The lumbar region of the spinal cord was isolated from control or treated mice at day 21 and 28 post-MOG immunization. Iba1 immunostaining (green) was performed to identify microglia/macrophages in the ventral white matter. To identify whether the microglia/macrophage cells were pro-inflammatory, iNOS (red) immunoreactivity was examined and to identify anti-inflammatory cells, Arg1 (red) was used. Nuclei were identified using DAPI (blue) **(A)**. Number of iNOS^+^Iba1^+^ or Arg1^+^Iba1^+^ cells were counted and divided by the total number of Iba1^+^ cells in the field to calculate a percentage **(B,C)**. Pro- to anti-inflammatory ratios were calculated by dividing the percent of iNOS1+ microglia by the percent of Arg1^+^ microglia **(D)**. Linear regression and comparison of slopes was performed on the day 21 and 28 averages to examine temporal regulation of microglia/macrophage polarization by the treatment groups **(E,F)**. Data are mean ± SEM. *n* = 4–6, *p < 0.05, **p < 0.01. ***p < 0.001 compared to control. Scale bar: 20 um.

Iba1 immunostaining was used to mark microglia/macrophages in the lumbar region of the spinal cord. Microglia present in control mice displayed an activated, ameboid phenotype throughout the disease course (Figures 4A, 5A). However, the microglia/macrophages in the combination-treated mice displayed a more complex, branched morphology, indicative of a less activated or anti-inflammatory phenotype (36) (Figures 4A, 5A). We performed a quantitative analysis of cell morphology to delineate these observed differences. There were no significant differences in cell body volume, number of processes emanating off the cell body, number of branch points, and average process length or volume (Figure S1). A slight trend toward increased branching and average process length and volume were noted in combination-treated mice. To parse out more subtle differences in morphology, Sholl analysis was performed (Figure 4B). Iba1^+^ cells from combination-treated mice had a significantly more complex branching structure compared to both untreated mice and the monotherapies at 10 and 15-microns from the cell body (Figure 4C).

The phenotype of microglia/macrophages was evaluated using immunofluorescence to examine colocalization of Iba1^+^ cells with inducible nitric oxide synthase (iNOS) to mark pro-inflammatory microglia/macrophages and Arginase-1 (Arg1) to mark anti-inflammatory cells (Figure 5A) (25, 32, 37). At the day 21 peak of inflammation, tuftsin alone and tuftsin together with benztropine significantly decreased the percent of Iba1^+^iNOS^+^ microglia/macrophages (Figure 5B). In untreated EAE animals, pro-inflammatory microglia/macrophages were typically present in clusters in the ventral white matter of the lumbar spinal cord, which is the area of the most immune-mediated damage in EAE (38, 39).

On the other hand, both control and treated EAE mice harbored high percentages of Arg1^+^ microglia/macrophages at day 21, whereas at day 28, the percent of anti-inflammatory cells in control mice was diminished, and mice receiving both drugs had a significantly higher percentage of Iba1^+^Arg1^+^ microglia/macrophages (Figure 5C). Since both untreated and benztropine-treated animals showed high percentages of iNOS^+^ microglia/macrophages and Arg1^+^ microglia/macrophages, there is likely global activation of these cells due to the absence of any immunomodulatory drug. When the pro- to anti-inflammatory microglial/macrophage cell ratio was calculated, only combination-treated EAE mice showed a significant decrease at the peak of the disease and a more modest decrease during recovery (Figure 5D).

We also examined how the temporal regulation of microglia/macrophage polarization changed between the peak and recovery time points in each treatment group (Figures 5E,F). Linear regression analysis showed that tuftsin, alone and in combination with benztropine, had significantly different slopes (reported in Table S1) of the change in percent iNOS^+^ microglia/macrophages compared to control. Typically, pro-inflammatory microglia/macrophages in untreated control mice do decrease from day 21 to day 28 in this model (25). This illustrates that immunomodulation is required to maintain low levels of pro-inflammatory cells throughout the disease course.

**Figure 6:**
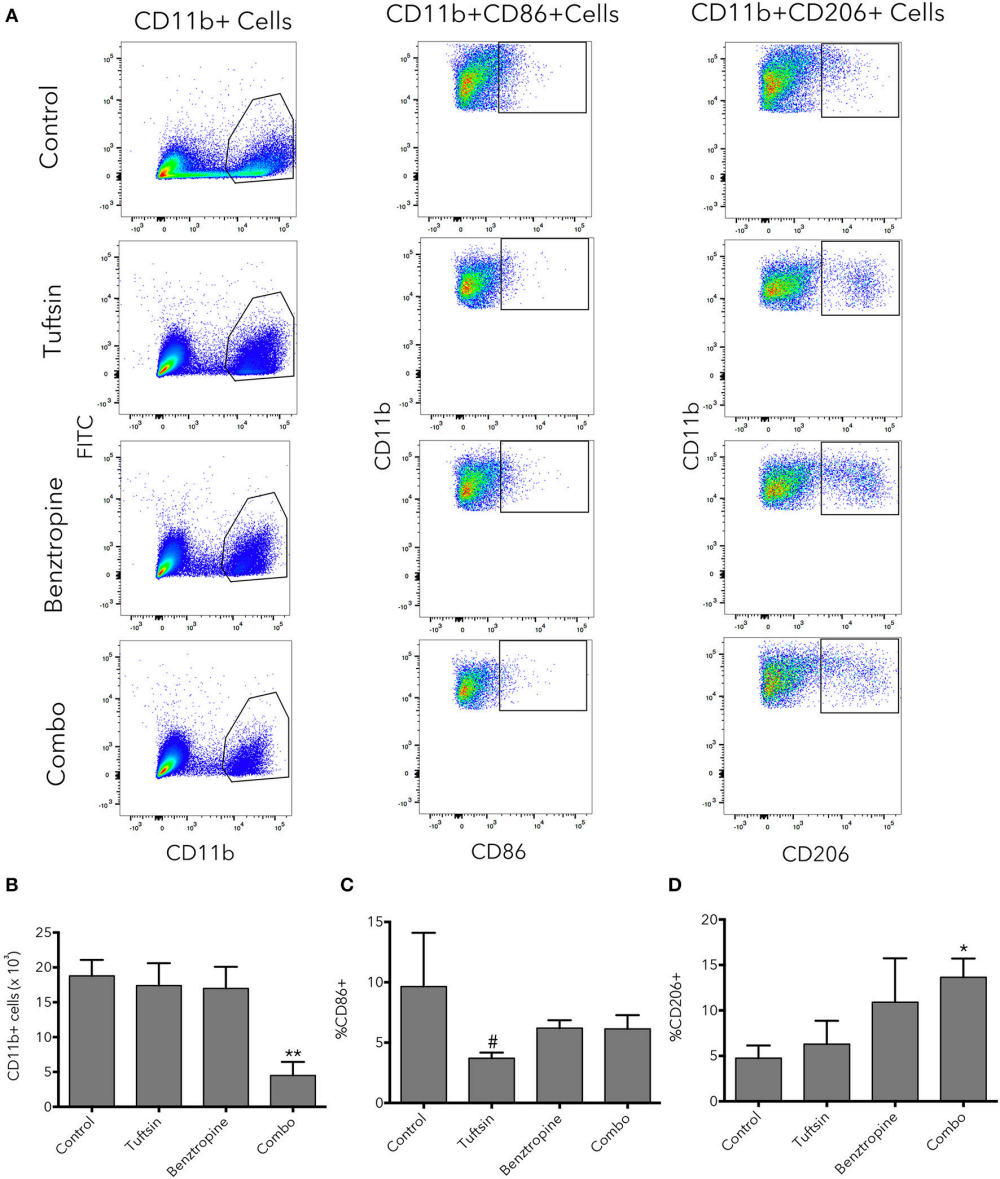
Combinatorial therapy significantly alters the number and phenotype of microglia/macrophages present in the spinal cord in EAE. At day 21 post-EAE induction, flow cytometry was performed on whole spinal cord lysates from control or treated mice. CD11b was used to identify the microglia/macrophage population. CD11b^+^CD86^+^ cells were considered of a pro-inflammatory nature and CD11b^+^CD206^+^ cells were considered anti-inflammatory **(A)**. Total CD11b^+^ cells **(B)** were plotted as well as percentage of CD11b^+^ cells that were CD86^+^ **(C)** or CD206^+^ **(D)**. Data are mean ± SEM. *n* = 3–4, *p < 0.05, **p < 0.01 compared to control. ^#^*p* < 0.05 compared to benztropine.

Flow cytometry was used as an additional method to evaluate the inflammatory state of microglial/macrophage phenotypes at the peak of the disease. An additional set of pro- and anti-inflammatory markers was utilized; CD11b was used to identify the microglia/macrophage population, CD86 as a marker of pro-inflammatory microglia/macrophages (40), and CD206 as a marker of anti-inflammatory microglia/macrophages (40, 41) (Figure 6A). Whereas, number of microglia/macrophages in untreated mice and mice treated with either drug alone was variable, mice treated with the dual therapy had significantly less CD11b^+^ cells present in the spinal cord compared to control mice, indicating less overall inflammation in the spinal cord (Figure 6B). Mice treated with tuftsin alone exhibited a significant decrease in pro-inflammatory CD86^+^CD11b^+^ cells compared to mice treated with benztropine alone (Figure 6C). CD206 expression in the CD11b^+^ population revealed that only the combinatorial treatment of tuftsin and benztropine had a significantly higher percentage of anti-inflammatory CD206^+^ microglia/macrophages compared to untreated mice (Figure 6D).

Taken together, immunofluorescent imaging and flow cytometric analysis indicate that benztropine may have a modest immunomodulatory effect on its own, but is ultimately not sufficient to fully alter the inflammatory CNS environment present in EAE toward one of an anti-inflammatory nature. However, upon combination with tuftsin, the CNS setting can be effectively modulated to dampen pro-inflammatory innate cell responses and promote anti-inflammatory phenotypes. In fact, this combination seems to produce the most beneficial and sustained effects in terms of inducing anti-inflammation compared to untreated animals.

### Benztropine is necessary to improve remyelination in the cuprizone model

**Figure 7:**
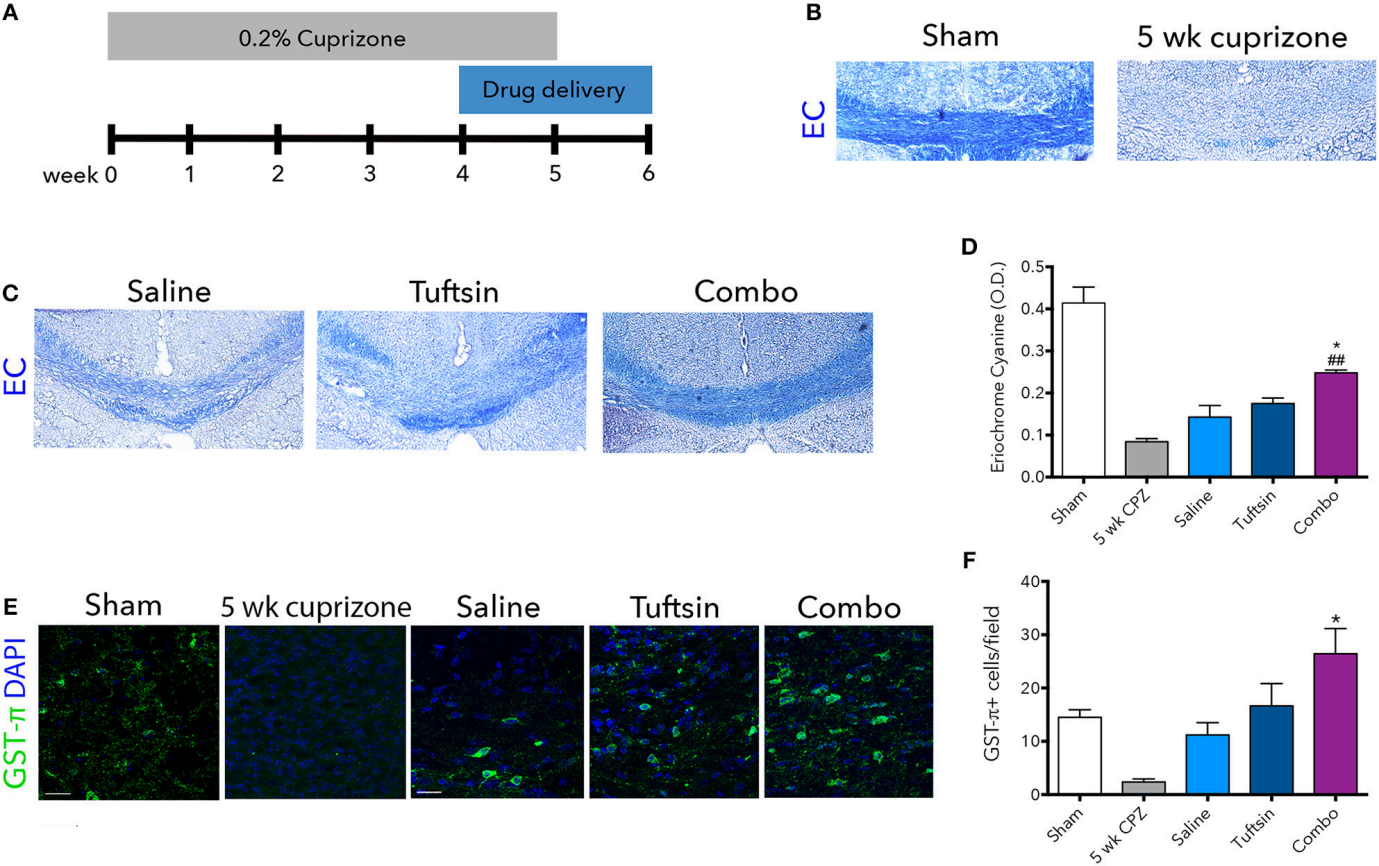
Tuftsin and benztropine improve remyelination in the cuprizone model. Mice were fed chow containing 0.2% cuprizone for 5 weeks. At week 4, mini-osmotic pumps containing saline, tuftsin alone, or both tuftsin and benztropine were inserted subcutaneously. Mice were reverted to standard chow to allow for 1 week of spontaneous remyelination in saline-treated animals **(A)**. At 5 weeks before withdrawal of cuprizone, a subset of mice were collected to assure demyelination in the corpus callosum with EC staining **(B)**. After 1 week of cuprizone withdrawal, saline-, tuftsin-, or combo-treated mice were collected and EC stain was used to evaluate degree of remyelination in the corpus callosum **(C)**. The optical density of the EC stain was calculated using ImageJ **(D)**. To visualize mature OLs, GST-π immunoreactivity (green) was examined **(E)**. Number of GST-π^+^ cells per field were quantified and plotted in **(F)**. Data are mean ± SEM. *n* = 2 (for 5 weeks cuprizone) – 3. Comparisons are between saline, tuftsin, and combo-treated mice. *p < 0.05 compared to control. ^##^*p* < 0.01 compared to tuftsin.

To examine remyelination in the absence of the complex inflammatory response in EAE, we utilized the cuprizone model of demyelination. Eight-weeks-old C57BL/6 mice were placed on a diet containing 0.2% cuprizone for 5 weeks to attain severe demyelination in the corpus callosum and a strong microglial response (42, 43). Mini-osmotic pumps were implanted in the mice containing saline, tuftsin alone, or tuftsin and benztropine together at week 4 of cuprizone administration, as remyelination occurs during the demyelination period as well (42). Mice were reverted back to normal chow at week 5 to allow for remyelination in the absence of demyelination for 1 week until histological analysis (Figure 7A). At 5 weeks, before withdrawal of cuprizone, almost complete demyelination was visualized by EC staining in the corpus callosum of untreated mice (Figure 7B). Remyelination was evaluated in the corpus callosum using EC 1 week post-cuprizone withdrawal (Figure 7C). Here, a significant increase in overall myelin was evident in the corpus callosum of combo-treated mice compared to those treated with either saline or tuftsin alone (Figure 7D).

To assess if this increase was due to the presence of more mature OLs through the established activity of benztropine (15), GST-π immunofluorescence was used to quantify OLs in the corpus callosum (Figure 7E). There were significantly more GST-π^+^ cells in mice treated with both tuftsin and benztropine compared to saline-treated mice (Figure 7F). This is consistent with benztropine enhancing OPC differentiation and thus, facilitating the remyelinating process. Therefore, the addition of benztropine was necessary to significantly improve remyelination 1 week post-cuprizone withdrawal.

### Tuftsin, alone or combined with benztropine, activates microglia/macrophages toward an anti-inflammatory state during the remyelination process

**Figure 8:**
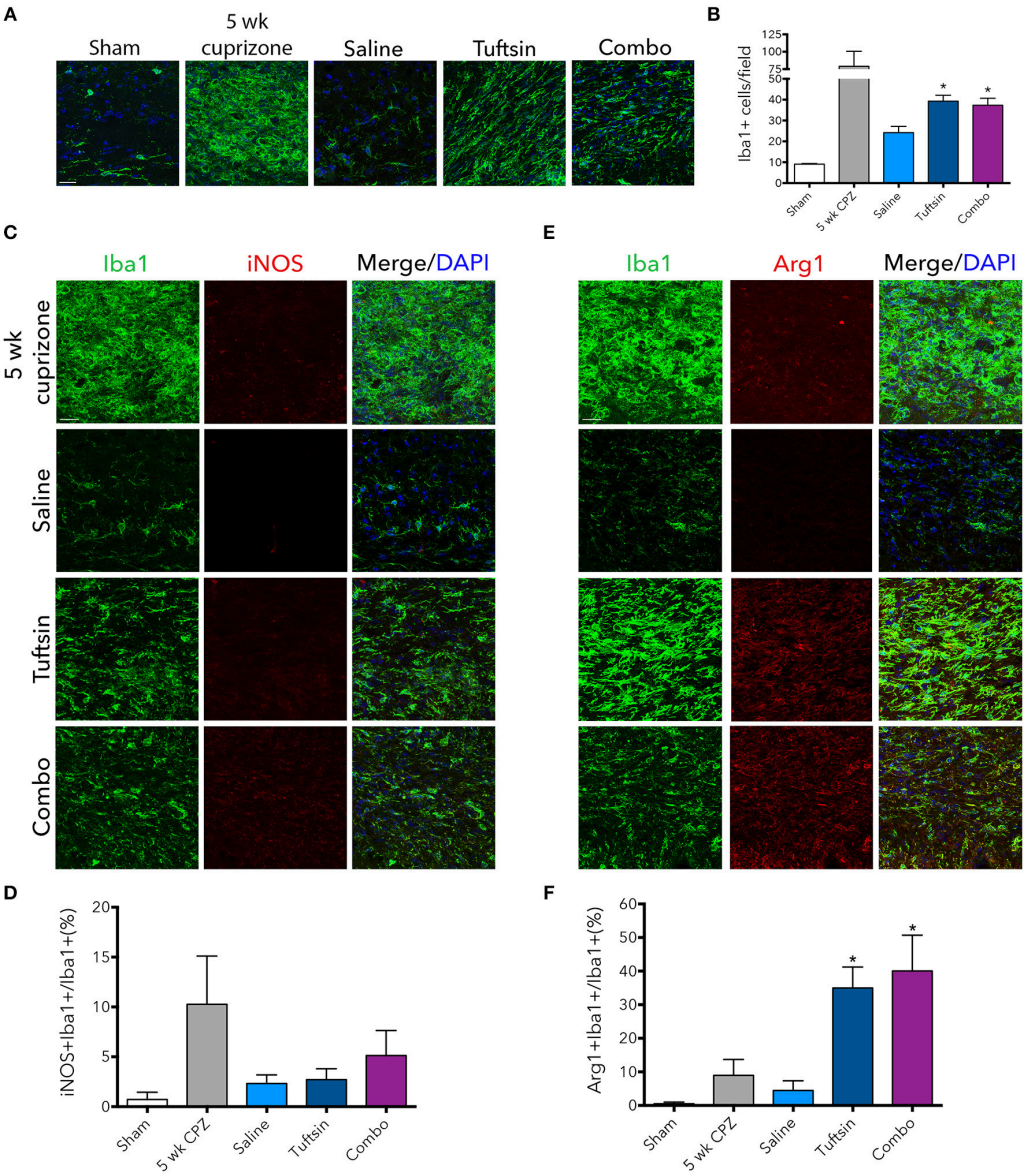
Tuftsin, alone or in combination with benztropine, promotes anti-inflammatory microglial activation in the cuprizone model during remyelination. One week after cuprizone withdrawal, brains were isolated from saline or treated mice. To visualize microglia/macrophages in the corpus callosum, Iba1 immunostaining (green) was performed **(A)**. Number of Iba1^+^ cells per field were quantified in **(B)**. To evaluate pro-inflammatory activation of microglia/macrophages in the corpus callosum, iNOS (red) immunoreactivity was examined **(C)** and quantified as percent of Iba1^+^ cells also expressing iNOS **(D)**. The same analysis was performed with Arg1 (red) to identify anti-inflammatory cells **(E,F)**. Data are mean ± SEM. *n* = 2 (for 5 weeks cuprizone) – 3. Comparisons are between saline, tuftsin, and combo-treated mice. *p < 0.05 compared to saline-treated mice. Scale bars: 20 um.

The numbers and phenotype of microglia/macrophages were assessed during remyelination after cuprizone-mediated demyelination. Iba1 immunofluorescence revealed significantly more microglia/macrophages in the corpus callosum in both tuftsin and combo-treated mice (Figures 8A,B). We also evaluated the phenotype of Iba1^+^ cells using iNOS to mark pro-inflammatory microglia, and Arg1 to mark anti-inflammatory microglia (Figures 8C,E). Whereas, cells from saline-treated mice expressed low levels of both markers indicating less activation (Figures 8D,F), mice treated with tuftsin, alone or together with benztropine, exhibited significantly more anti-inflammatory, Arg1^+^Iba1^+^ cells in the corpus callosum (Figure 8F). There were no significant differences in iNOS^+^ cells (Figure 8D). Taken together, these results indicate that administration of tuftsin in the cuprizone model of demyelination activates microglia/macrophages in an anti-inflammatory manner, whether administered alone or together with benztropine. However, microglial/macrophage modulation by tuftsin alone was not sufficient to improve remyelination in this non-immune cell-mediated model.

## Discussion

In this study, we demonstrate that tuftsin, a microglia/macrophage-targeting immunomodulatory tetrapeptide (23–26), in conjunction with benztropine, an inducer of OPC differentiation shown to facilitate remyelination (15), is effective in two animal models of MS: EAE and cuprizone-mediated demyelination. We employed the two reagents together, therapeutically, in both models in order to modulate the cell types affected by each individual reagent after initiation of MS-like pathological processes. Our goal was to transform the inflammatory CNS environment, which is normally present in MS, into one of an anti-inflammatory nature permissive to the preservation of myelin and myelin repair by employing the drugs in the EAE model, as well as study their combined action in cuprizone, a model in which remyelination can be more clearly evaluated.

In EAE, therapeutic, conjunctive administration of tuftsin with benztropine significantly decreased demyelination, attenuated inflammation, and promoted anti-inflammatory responses of microglia and infiltrating macrophages in the spinal cord throughout the duration of the disease. Although administration of these drugs as monotherapies was effective at reducing motor deficits and attenuating the agents' respective targeted pathologies at the peak of EAE, the combination treatment produced the most significant results throughout the disease course.

Regarding the symptomatic readout of the EAE model employed, our data show that the combination treatment significantly improved disease scores compared to control mice as well as compared to mice treated with benztropine alone at the peak of the disease. Thus, in the therapeutic treatment timeline used, even with benztropine acting to induce OPC differentiation (Figure 3D), OLs were likely unable to keep up with the high demand for repair at the point of the disease when highest inflammation is observed. The intense inflammatory environment in MS/EAE has previously been reported to impair OL function due to endoplasmic reticulum stress and high levels of reactive oxidant species (ROS) (44).

When examining demyelination, a major pathological hallmark of MS/EAE, we observed that both benztropine- and combination-treated mice had notably less demyelination at day 21 compared to control mice, but only the combination therapy significantly improved this measure at day 28. Though tuftsin-only treatment trended toward less demyelination, it was surprising that it didn't significantly reduce demyelination on its own, as we have previously reported (25, 26). However, in prior studies, tuftsin administration was initiated on the same day as EAE induction or only 7-days post-MOG immunization (23, 25), whereas in the current study we started drug infusion 14-days post-induction when mice had scores ranging from 0.5 (initial loss of tail tone) to 1.5 (completely limp tail with loss of feeling in hind limbs). This result illustrates that on a more relevant therapeutic timescale, tuftsin-mediated modulation of microglia and macrophages alone doesn't mitigate demyelination in this model. This also demonstrates that the dual treatment of tuftsin and benztropine, administered together at a therapeutic time point, is effective at ameliorating demyelination throughout the entire disease course.

Since cells of the oligodendrocyte lineage are responsible for the process of remyelination and benztropine targets OPC differentiation (15), we examined numbers of NG2^+^ OPCs and CC1^+^ OLs in each treatment group. OPCs respond to inflammation with increases in reactivity characterized by morphological changes and intense immunoreactivity with NG2, however, they have only been reported to proliferate when inflammation is accompanied by demyelination (5, 45). Here, we did not see any significant changes in number of NG2^+^ OPCs or CC1^+^ OLs between treatments, though differences in reactivity of NG2^+^ cells were apparent when inflammation was uncontrolled in the untreated and benztropine-treated groups. There was no significant increase in mature OLs evident by CC1 or GST-π analysis, which was surprising as it was previously reported to increase GST-π^+^ cells in the spinal cord of EAE mice (15). However, in those experiments a relapsing-remitting disease course was induced using proteolipid protein (PLP) immunization, whereas we utilize MOG immunization in our EAE model. There was, however, a significant increase in the CC1^+^/NG2^+^ cell ratio in mice treated with benztropine alone at the inflammatory peak of the disease. And though the ratio of these cells from combination-treated mice was variable, it did show a trend toward more OLs compared to OPCs at both time points, whereas the untreated and tuftsin-treated mice maintained lower CC1^+^/NG2^+^ cell ratios. This paralleled the demyelination data, where benztropine resulted in significantly less white matter demyelination at day 21, and combination-treated mice were significantly less demyelinated at both day 21 and 28 (Figure 2B).

Lastly, we examined microglial/macrophage activation and phenotypes, and beneficial immunomodulatory effects were observed in the EAE mice treated therapeutically with both tuftsin and benztropine. Though it is now understood that microglia and macrophages can acquire a heterogeneous and complex array of phenotypes, it has been shown that these cells predominantly display a more pro-inflammatory phenotype during the peak of MOG-induced EAE and during relapses in models of RRMS (25, 33). It is favorable to increase the number of anti-inflammatory microglia at this point, as they are able to contribute to repair processes (26, 33, 40, 46). Flow cytometric analysis at the height of the disease course illustrated a significantly higher percentage of anti-inflammatory CD206^+^ microglia and significantly lower overall microglial/macrophage numbers in the combination treated mice compared to untreated EAE mice (Figures 6B,D). Thus, tuftsin together with benztropine not only limits the recruitment of immune cells, but assures that the cells present are of a more anti-inflammatory nature as we have previously seen with prophylactic treatment with tuftsin alone (25, 26). In addition, immunofluorescent analysis showed that the combination treatment drastically decreased the percent of pro-inflammatory microglia/macrophages at the peak of the disease, as evaluated by iNOS levels, and boosted anti-inflammatory (Arg1^+^) microglia/macrophage populations significantly during the recovery phase, thereby addressing their activation throughout the entire disease course. It was surprising that our results differed between the immunofluorescent analysis where we observed that combination therapy decreased pro-inflammatory microglia/macrophages (Figure 5) and flow cytometry, where tuftsin and benztropine together increased anti-inflammatory microglia/macrophages, but did not significantly decrease pro-inflammatory cells (Figure 6). This is likely due to the difference in markers used in each technique as well as the fact that immunofluorescent analysis was performed at specific regions of the ventral white matter in the lumbar spinal cord, whereas flow cytometry was done on whole spinal cord lysate.

At the peak of the disease, benztropine-only treatment also modestly shifted microglial phenotypes away from pro-inflammatory, though not significantly. This was surprising since benztropine and other muscarinic antagonists, like clemastine, were previously observed to have no immunomodulatory effects in EAE (15, 17). However, this could be due to indirect effects on microglia and macrophage recruitment and phenotypes through the promotion of OPCs, as OPCs (but not OLs) have been reported to express CCL2 and IL1β, which act as chemoattractants for immune cells (47). IL1β is also a pro-inflammatory cytokine that is found at high levels in CNS lesions of MS patients and has been shown to contribute to EAE pathogenesis (48). It is possible that as benztropine shifts OPCs toward OLs, it may be resulting in decreases in the expression of these recruitment factors, thereby moderately reducing inflammation on its own. There is also a small subpopulation of microglia that do express the M3 muscarinic acetylcholine receptor, to which benztropine binds (49). In models of other neurodegenerative conditions, such as Alzheimer's and stroke, this M3-positive microglial population expands and has functional reactions to receptor stimulation, such as increased chemotaxis and decreased phagocytic activity, however, in EAE these microglia do not expand their functional responses to receptor stimulation (49). Thus, this fact along with previously established lack of immunomodulation in various EAE models indicate that it is not likely that benztropine exerts a direct effect on microglia.

Though remyelination is evident in EAE (17, 50), the inflammatory environment can complicate the study of this process and, as our data shows, the mechanism of remyelinating agents can be difficult to discern in this model. Thus, demyelination induced by toxins, such as cuprizone or lysolecithin, is a better method to evaluate reparative mechanisms (43). Here, we applied the cuprizone model and report that the addition of benztropine to tuftsin resulted in improved remyelination in the corpus callosum 1 week after cuprizone withdrawal, likely due to increased presence of mature OLs (Figures 7C–F). Benztropine alone has previously been seen to enhance myelination in this model by increasing OL numbers in the corpus callosum, though those differences were observed 2 weeks after reverting back to normal chow (15), whereas we examine 1 week post-cuprizone withdrawal.

Tuftsin alone did not significantly affect remyelination or number of GST-π^+^ OLs in the corpus callosum of cuprizone-treated animals, however, the numbers of GST-π^+^ OLs were marginally increased compared to saline-treated animals. Several studies have shown that microglia are critical facilitators in the remyelinating process and that anti-inflammatory phenotypes can drive OPC differentiation and promote regeneration (51, 52). In fact, our results do show that tuftsin (alone and together with benztropine) promote anti-inflammatory microglial/macrophage activation the cuprizone model (Figures 8E,F). Microglia with an overall anti-inflammatory phenotype, as is induced by tuftsin (23–26), are reported to more efficiently phagocytose and clear myelin debris, a major obstacle to remyelination (53), as well as express factors that recruit and promote differentiation of OPCs, such as IGF-1 and TGF-β (52, 54). However, this indirect mechanism of modulating OPCs through the modulation of microglia and macrophages may not be as potent or rapid as the direct effects of benztropine. It is possible that extending the time allowed for remyelination by another week may amplify tuftsin's effects when administered alone.

Overall, our data show that combining tuftsin with benztropine improves pathologies in two MS models, one which underlines inflammation in the CNS and the other which allows for the study of the remyelinating process. In EAE, the combination therapy consistently results in reduction of clinical scores and inflammatory pathologies at multiple time points, suggesting the creation of an environment that may prevent demyelination and OL damage, and will push microglia and macrophages toward a phenotype that is more conducive to remyelination and repair. In the cuprizone model, the combination therapeutic treatment significantly improved remyelination over saline or tuftsin treatment alone. These results are reflective of the inherent strengths and limitations of each model, with immunomodulation revealing importance in EAE and OPC manipulation crucial to improvement in cuprizone. We are currently investigating the benefits of similar therapies, which target anti-inflammatory and remyelinating mechanisms, in the models utilized in this study as well as other translationally-relevant EAE models. Specifically, the PLP-induced model of RRMS that simulates multiple relapses (55, 56), or a more severe and progressive model, such as that induced by MP4, a myelin basic protein-proteolipid protein fusion peptide (57) or MOG immunization in DBA/1 mice (36, 58, 59), will likely show more dramatic differences between monotherapies and dual therapy and allow us to discern more temporal effects. Even as promoting remyelination emerges as a viable and potentially symptom-reversing therapeutic strategy, immune modulation will remain critical and our study shows that tuftsin works effectively in combination with benztropine in two primary MS models.

## Data availability statement

The raw data supporting the conclusions of the manuscript will be made available by the authors, without undue reservation, to any qualified researcher.

## Author contributions

KT designed and performed experiments, analyzed the data and wrote drafts of the manuscript. JN performed experiments and analyzed the data. AP analyzed data. ST designed experiments, analyzed data, and wrote drafts of the manuscript.

### Conflict of interest statement

The authors declare that the research was conducted in the absence of any commercial or financial relationships that could be construed as a potential conflict of interest.

## Abbreviations

CFA: complete Freund's adjuvant
CNS: central nervous system
EAE: experimental autoimmune encephalomyelitis
FDA: Food and Drug Administration
MOG: myelin oligodendrocyte glycoprotein
MS: multiple sclerosis
Nrp1: neuropilin-1
OPC: oligodendrocyte precursor cell
OL: oligodendrocyte
PPMS: primary progressive multiple sclerosis
ROS: reactive oxidant species
RRMS: relapsing-remitting multiple sclerosis
SPMS: secondary progressive multiple sclerosis
Treg: T-regulatory cell.
