# Supplementary material for: Tuftsin Combines With Remyelinating Therapy and Improves Outcomes in Models of CNS Demyelinating Disease
Source: Front Immunol. 2018 Nov 28;9:2784. doi: 10.3389/fimmu.2018.02784 (PMC6283261; doi:10.3389/fimmu.2018.02784)
Supplement: Supplementary file 1 [file Data_Sheet_1.PDF]

Supplementary Material

Tuftsin combines with remyelinating therapy and improves outcomes in models of CNS demyelinating disease

Kaitlyn K. Thompson[1]; Jillian C. Nissen[1,#], Amanda Pretory[1], *Stella E. Tsirka[1]

[1]Program in Molecular and Cellular Pharmacology, Department of Pharmacological Sciences, Stony Brook University, NY, USA

***Correspondence:**

Dr. Stella E. Tsirka, Department of Pharmacological Sciences, Stony Brook University, NY 11794-8651; Tel: 631-444-3859; Email: styliani-anna.tsirka@stonybrook.edu

1 Supplementary Figures and Tables

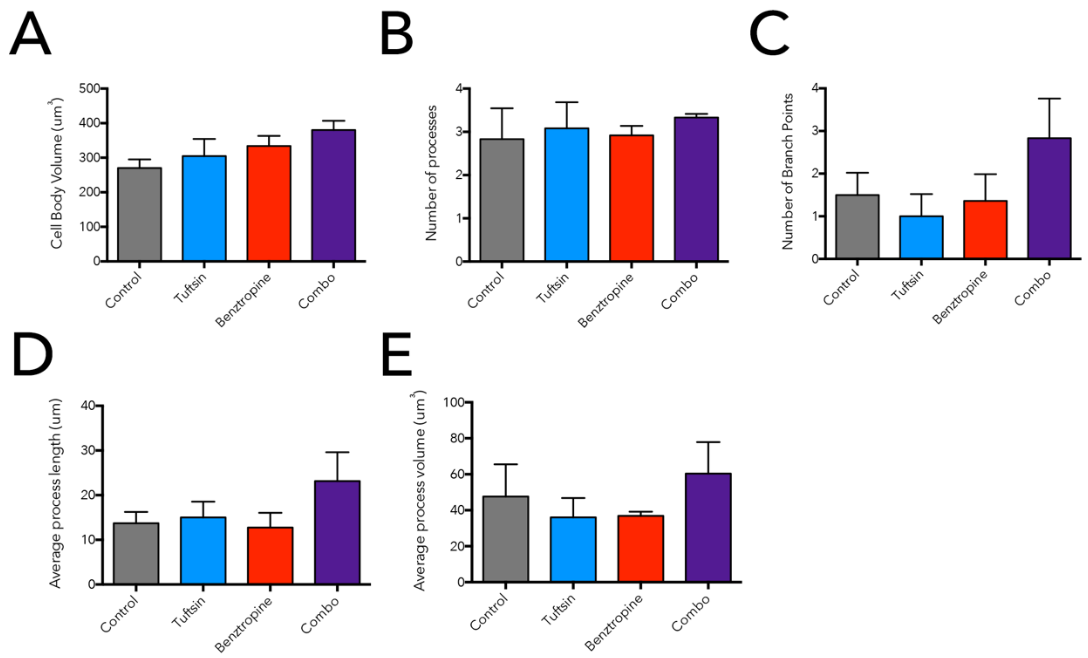

Figure S1. Microglial morphometrics did not change significantly upon treatment with tuftsin, benztropine, or both. 20 micron frozen lumbar spinal cord sections were immunostained with Iba1 and imaged at 63x on a confocal microscope. Cell structures completely within the set x, y, and z parameters were traced using Neurolucida software. 3D reconstructions were created in Neurolucida Explorer and parameters were measured using this software. Measures include: cell body volume (A), number of processes off cell body (B), number of branch points (C), average process length (D), and average process volume (E). Data are mean ± SE. n = 3 biological replicates, 5 cells/replicate.

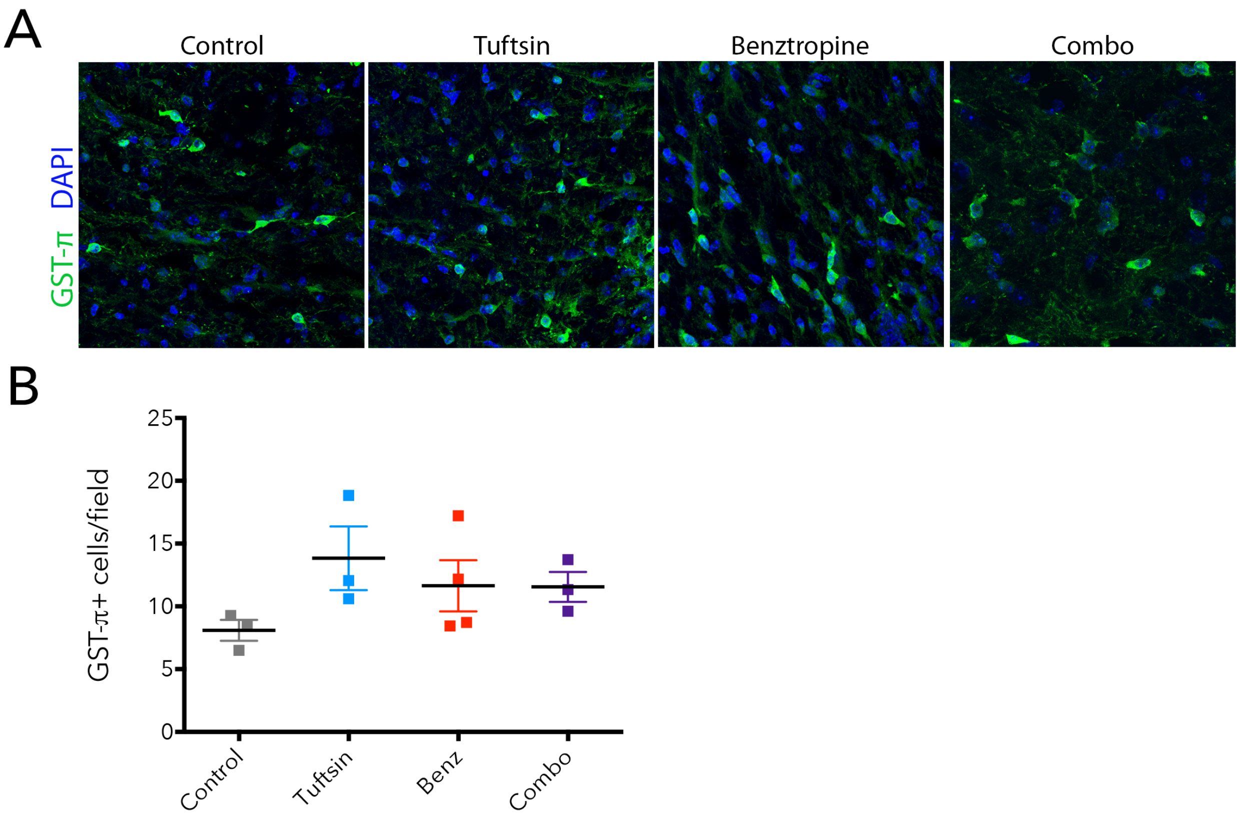

Figure S2. Mature oligodendrocytes at Day 21 post-MOG immunization. 20 micron frozen lumbar spinal cord sections were immunostained with GST-π and imaged at 63x on a confocal microscope (A). GST-π+ cells were quantified (B). Data are mean ± SE. n = 3.

Treatment	iNOS Slope	Arg1 Slope
Control	-7.434 ± 1.825	-4.091 ± 1.336
Tuftsin	-1.293 ± 1.974	-3.381 ± 2.547
Benztropine	-1.320 ± 2.672	-1.864 ± 2.500
Combo	0.3581 ± 0.6333	-4.130 ± 2.553

Table S1. Linear regression slopes for iNOS+ and Arg1+ microglia and macrophages from Day 21 to 28. Mean percent of microglia/macrophages expressing iNOS or Arg1 was plotted (Figure 5E & 5F) and linear regression analysis was performed. Best-fit values ± SEM are reported.
